# Physical activity promotion for patients transitioning to dialysis using the “Exercise is Medicine” framework: a multi-center randomized pragmatic trial (EIM-CKD trial) protocol

**DOI:** 10.1186/s12882-018-1032-0

**Published:** 2018-09-12

**Authors:** Ram Jagannathan, Susan Lynn Ziolkowski, Mary Beth Weber, Jason Cobb, Nhat Pham, Jin Long, Shuchi Anand, Felipe Lobelo

**Affiliations:** 1 Hubert Department of Global Health, Rollins School of Public Health, Emory University, 1518 Clifton Road NE, CNR 7051, Atlanta, GA 30322 USA; 2 Division of Nephrology, Stanford University School of Medicine, Palo Alto, CA USA; 3 Renal Division, Department of Medicine, Emory University School of Medicine, Atlanta, Georgia USA; 4 Division of Nephrology, Santa Clara Valley Medical Center, San Jose, CA USA; 5 Exercise is Medicine Global Research and Collaboration Center, 1518 Clifton Road NE, CNR 7051, Atlanta, GA 30322 USA

**Keywords:** Chronic kidney disease, Exercise is medicine, Intervention, Physical activity, Qualitative interviews, Estimated glomerular filteration rate

## Abstract

**Background:**

Patients on dialysis are physically inactive, with most reporting activity levels below the fifth percentile of healthy age-matched groups. Several small studies have reported efficacy of diverse exercise interventions among persons with CKD and those on dialysis. However, no single intervention has been widely adopted in real-world practice, despite a clear need in this vulnerable population with high rates of mortality, frailty, and skilled nursing hospitalizations.

**Methods/design:**

We describe a pragmatic clinical trial for an exercise intervention among patients transitioning to dialysis. We will use an existing framework – Exercise is Medicine (EIM) – developed by the American College of Sports Medicine. After undertaking formative qualitative research to tailor the EIM framework to the advanced CKD population (eGFR < 30 ml/min/1.73m^2^), we will randomize 96 patients from two regions—Atlanta and Bay Area—in two intervention arms with incremental levels of clinical-community integration: physical activity assessment during Nephrology clinical visit, brief counseling at pre-dialysis education, and physical activity wearable (group 1) versus group 1 intervention components plus a referral to a free, EIM practitioner-led group exercise program over 16 weeks (group 2; 8 week core intervention; 8-week follow up). We will assess efficacy by comparing between group differences in minutes/week of objectively measured moderate intensity physical activity. To evaluate implementation, we will use questionnaires for assessing barriers to referral, participation and retention along the path of the intervention. Further we will have a plan for dissemination of the intervention by partnering with relevant stakeholders.

**Discussion:**

The overall goal is to inform the development of a practical, cost-conscious intervention “package” that addresses barriers and challenges to physical activity commonly faced by patients with advanced CKD and can be disseminated amongst interested practices.

**Trial registration:**

ClinicalTrials.gov identifier (Dated:10/17/2017): NCT03311763.

## Background

In 2017, 30 million adults or 15% of the United States population were estimated to have chronic kidney disease (CKD) [1]. It is well reported that minorities [2] have a higher reported incidence of CKD and end stage renal disease (ESRD), with African Americans and Hispanics having 3.9 and 1.5 (respectively) higher odds to develop ESRD compared to whites [3, 4]. Despite medical advances, CKD remains a debilitating disease with a myriad of understudied symptoms and consequences, including insomnia [5], depression [6, 7], low muscle mass [8, 9], and low physical activity (PA) levels [10–13], all contributing to substantial decrements in quality of life and adverse medical outcomes [7]. Nearly 95% of persons newly starting dialysis have physical fitness levels below the 20th percentile for the general population, and only 56.4% are reportedly able to walk one block [11]. Functional decline only accelerates after start of dialysis and leads to a negative spiral of deconditioning [14].

Prospective studies on patients with CKD requiring dialysis have shown a 60% or higher risk for mortality among those with the lowest level of PA compared to those with average or above average activity and/or fitness levels [13, 15, 16]. Further, sedentary behavior is associated with an increased risk for mortality among dialysis patients similar in magnitude to that of other well-established risk factors, such as a one-point reduction in serum albumin concentration [13]. Given the high cardiovascular mortality in persons with advanced CKD [17, 18] and the limited treatments available to patients who progress to end-stage kidney disease, there is a need to focus on promoting PA, a modifiable risk factor with potential for huge improvements in cardiovascular mortality, quality of life, and symptoms of uremia, which may improve tolerance dyalisis while countering the natural history of accelerated functional decline typically experienced by these patients [19]. The 12 month period preceding the anticipated start of dialysis may therefore be an important window of opportunity to implement PA interventions in this clinical population.

Previous intervention studies in persons with advanced CKD or those on dialysis have shown improvement in physical performance [20, 21], health-related quality of life [21–23], and muscle strength or quality [20, 24, 25] with exercise therapy. Additional studies provide evidence for anti-inflammatory effects of exercise training among persons with advanced CKD [26]. The interventions described in these studies have differed in many aspects: setting (in dialysis center versus home based), intensity (prescribed versus patient driven), and type (aerobic, resistance, or combined) [27]. Despite these efforts, no single intervention design has emerged as a sustainable standard of care model for nephrology practices interested in implementing standardized PA programming as part of CKD management.

For the present study, we plan an exercise intervention that optimizes use of existing resources to integrate PA in the management of patients with advanced CKD transitioning to dialysis, with the main purpose of creating a transferrable intervention “package” that acknowledges and tackles implementation barriers in clinical and community settings. For this, we adapted the Exercise is Medicine (EIM) Framework, an initiative conceived in 2007 by the American College of Sports Medicine (ACSM) and co-launched by the American Medical Association, with the ultimate mission of implementing evidence-based PA intervention as standard of care for every patient [28]. This includes approaches for routinely integrating PA assessment, counseling and prescription/referral programs, particularly for patients with chronic diseases [29]. Herein, we describe a protocol to adapt the EIM framework for integrating PA promotion among patients transitioning to dialysis, integrating in-depth interviews with patients with CKD and feedback from community partners and directly test, in a pragmatic trial, the feasibility and short-term impact of this intervention on PA levels and secondary outcomes.

## Methods

### Research rationale and objectives

**Fig. 1:**
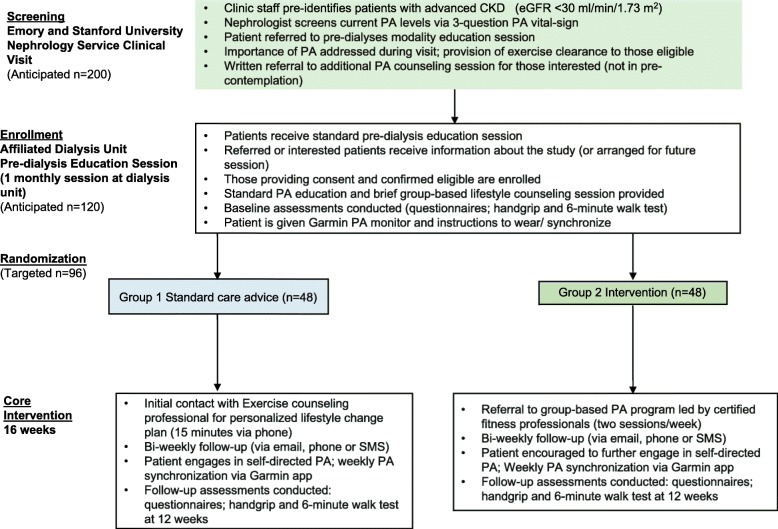
Study design. Questionnaires: Demographics, age, sex, race, ethnicity, education, if employed/actively working, cause of CKD, vascular access yes/no, other comorbidities, medications; Symptoms of Depression (CEDS-20), SF-12, Physical activity self-efficacy questionnaires; *CKD* Chronic Kidney Disease, *PA* Physical activity’

The overall goal of this research is to develop and validate a pragmatic and scalable intervention, working with patients and community partners to adopt the existing EIM framework to improve PA levels among patients with advanced CKD but not yet in dyalisis (Fig. 1). We have three main objectives:

1. Develop a framework for a PA intervention, seeking input from patients using a qualitative methodology
2. Synthesize and use the findings from Aim 1 to implement a PA intervention and test it in a randomized controlled pragmatic trial (8-week intervention and 8 week follo-up periods) in two settings enriched with minority populations.
3. In addition to assessing short term effectiveness of the intervention, focus on implementation by assessing the barriers to referral, participation, and retention along the path of the planned intervention and on future scalability by engaging relevant stakeholders.

### Exercise is medicine framework

The EIM Framework serves as the backbone for this study (www.exerciseismedicine.org) https://www.quantextual.co/researcher/. This framework calls for routine assessment of PA during clinical interactions (called the “physical activity vital sign”), brief PA counseling, and a specific written PA prescription and/or referral to a community-based PA program, professional or resource. The PA prescription is often supplemented with referral to certified EIM practitioners (i.e. fitness professionals) that can lead exercise sessions, often group-based, tailored to specific chronic diseases. A feedback mechanism from the EIM practitioner to both the patient and to the physician is also recommended, often using PA wearable monitoring technologies [30]. An evaluation framework for EIM implementation in health care settings has also been published [31].

### Research setting and team

Participants for both the formative qualitative work and the intervention will be recruited through recommendation and referral from treating physicians at a Bay Area (Santa Clara Valley Medical Hospital Nephrology Clinic, an affiliate of Stanford University) and Atlanta (Emory Nephrology Clinic, Emory University). A group of seven nephrologists staff the Santa Clara Valley Medical Hospital Kidney clinic, serving a clinic population primarily insured under the county health plan and enriched with patients of Hispanic/Latino and Southeast Asian descent. Emory University’s Midtown Kidney Clinic staffs seven academic nephrologists, and services a clinic population enriched with African American patients. In addition to the research teams and clinicians, fitness professionals certified in the Exercise is Medicine requirements https://www.quantextual.co/researcher/ will be recruited at each site to help lead PA counseling and group exercise sessions. The trial is prospectively registered on clinicaltrials.gov (NCT03311763) as of October 17, 2017.

### Participant recruitment

The study design and inclusion/ exclusion criteria are shown in Table 1. English-speaking not yet on dialysis patients been seen at the two mentioned study clinical sites will complete a screening PA vital sign questionnaire, and thereafter study research coordinators will assess him or her for eligibility based on pre-specified inclusion/ exclusion criteria. Additionally, nurse recommendations and fliers briefly outlining the study posted within the kidney clinics will be used to help identify prospective participants. Potentially eligible patients will be contacted face-to-face or via telephone by a member of the research team to provide additional details on the study and its objectives. If ongoing interest is expressed, we will seek approval from nephrologists prior to enrolling. Research coordinators will obtain written consent from patients willing to participate in the trial.

**Table 1:** Inclusion and exclusion criteria

Inclusion criteria • Not yet on dialysis, but eGFR < 30 ml/min/1.73m^2^ • Age ≥ 30 and ≤ 80 years • Self-reported Physical activity < 30 min/week of moderate intensity physical activity • Non-wheelchair bound • Able to provide informed consent in English • Anticipated to be living in the study area during the study period Interested in becoming more physically active over the next 6 months Exclusion Criteria • Inability to provide consent in English • Diagnosed mental health disorder • Alcohol or drug abuse • No fixed address or contact details • Unstable angina or unstable arrhythmias • Lack of access to internet • Any concern not otherwise stated by patient’s nephrologist	

### Formative data collection and intervention development

For the tailoring of the EIM framework for patients with advanced CKD, we will use (1) feedback from community partners and (2) in-depth interviews with patients with CKD at the intervention site. We aim to interview a total of 20 participants (10 per site), stratified by sex (male/female) and diabetes status (yes/NO) for a total of five patients in each sex-diabetes status strata. Participants will be asked to participate in a 1-h, semi-structured interview to discuss their exercise views and experiences, impact of CKD in exercise behaviors, opinions on planned intervention components (e.g., exercise classes, group-based exercise, mobile phone applications), and measures of programmatic success. Interviews will be digitally recorded using a smart phone equipped with specialized applications (Pocket Dictate for iPhone and Express Dictate for Andriod). Audiofiles will be transcribed verbatim for analysis. Transcripts will be reviewed and iterative processes will be employed to update the interview guide to add additional probes to gather more information or update questions if saturation is reached on a particular issue. An initial codebook including both deductive and inductive codes will be developed after reviewing all of the transcripts, and this codebook will be finalized through consultation with the study investigators. We will then conduct a thematic analysis, using MaxQDA (Verbi software) to assist with data manipulation and with structured comparisons of the data (men vs. women, individuals with and without diabetes, participants at each site) around key themes, to describe exercise behaviors and program recommendations from potential program users. The results of the analysis will then be applied to the EIM framework to finalize the intervention design.

### Baseline data collection

Data collection will occur at baseline and at 8- (V1) and 16 weeks (V2) after the interventions (Table 2). Personal information, including relevant medical history will be collected prior to the initiation of testing or intervention upon receiving participant consent. This will include patient characteristics of age, sex, basic anthropometric measures (height, weight, BMI and waist circumference), relevant comorbidities and medications.

**Table 2:** Interventions examples

Class format	Minutes	Teachable moments
a. Strong bones and healthy hearts		
Warm-up and rhythmic stretch	10–12	• Pre-Warm-Up: Perceived exertion, breathing and hydration. • Action Planning: ROM safety through major/minor joints. • Warm-up: Large to small muscles.
Circuit Choreography	15	• Selecting the right tubing resistance, benefits of strength work, benefits of cardio work & the plus for both in a circuit format. • Resistance tubing safety. • Breathing techniques for strength work. • Cardio-cognitive benefits • Benefits of tempo changes through half-time, at music tempo and double- time tempos. • Benefits of non-dominant side movements.
Cool-Down	5	• Benefits of lowering the heart rate progressively.
Core Strengthening	5	• Benefits of concentric, eccentric strength training.
Flexibility Training	5–8	• Option - Chair transfer technique from chair to floor and from floor to chair. • Range of Motion versus Range of Movement.
Relaxation & Future Action Planning	5–10	• Breaking the symptom cycle and self-managing pain, fatigue, frustration, etc.
b. Stability & Balance for Fall Prevention		
Warm-up and rhythmic stretch		• Pre-Warm-Up: Perceived exertion, breathing and hydration. • Action Planning: ROM safety through major/minor joints. • Warm-up: Large to small muscles.
Core stability and strength training	5	• The muscle groups which compose the core. • Benefits of breathing for increasing abdominal chest wall strength and flexibility
Balance training	5–10	• Agility – changing center of gravity • Lower body strength
Stability training	5–10	• Stability in an unstable environment • How to protect the lower back during extensions • PNF stretching principles
Flexibility	10	• Stretch tight muscles for better posture • Use breathing to deepen stretches
Relaxation and Action planning	10	• Breaking the symptom cycle to better self-manage pain, fatigue, fear, anger, frustration and physical instabilities

### Randomization

Participants will be randomized to group 1 (low intensity) and group 2 (full –scale) intervention groups. Randomization will be conducted at a participant level prior to baseline testing by blocks of 16 via a computer-generated random number sequence by an independent researcher. Blinding of intervention group to clinical staff and participants will not be possible once the intervention starts.

## Trial interventions

### Group 1 and 2

#### Brief physical activity counseling

After study enrollment and assessment of the PA vital sign, a 30-min PA counseling session will be provided by the EIM – fitness professional. Evidence suggests that interventions should address individual, social and environmental factors to improve the likelihood of influencing behavior [32, 33]. Socio-ecological models of PA conceptualize and address the interaction and interdependency between individual, social and environmental factors [34, 35] and help identify the different environments where PA can be promoted, [36]. Health care and community settings are places where large proportions of the population can be reached and where PA can be effectively promoted [37–41]. By addressing multi-level influences for PA, the EIM intervention is rooted on socio-ecological approaches to increase the impact and sustainability of the intervention [33].

The PA counseling session will use brief-action planning, a highly structured, self-management support technique grounded in motivational Interviewing and behavior change strategies (goal setting, identify preferences and barriers, problem solving, stages of change and self-monitoring). Patients will be encouraged to incorporate moderate PA throughout the week to achieve 100–150 min/weekly, primarily through walking for leisure and transportation or other preferred activities [42].

#### Werable physical activity monitor

**Fig. 2:**
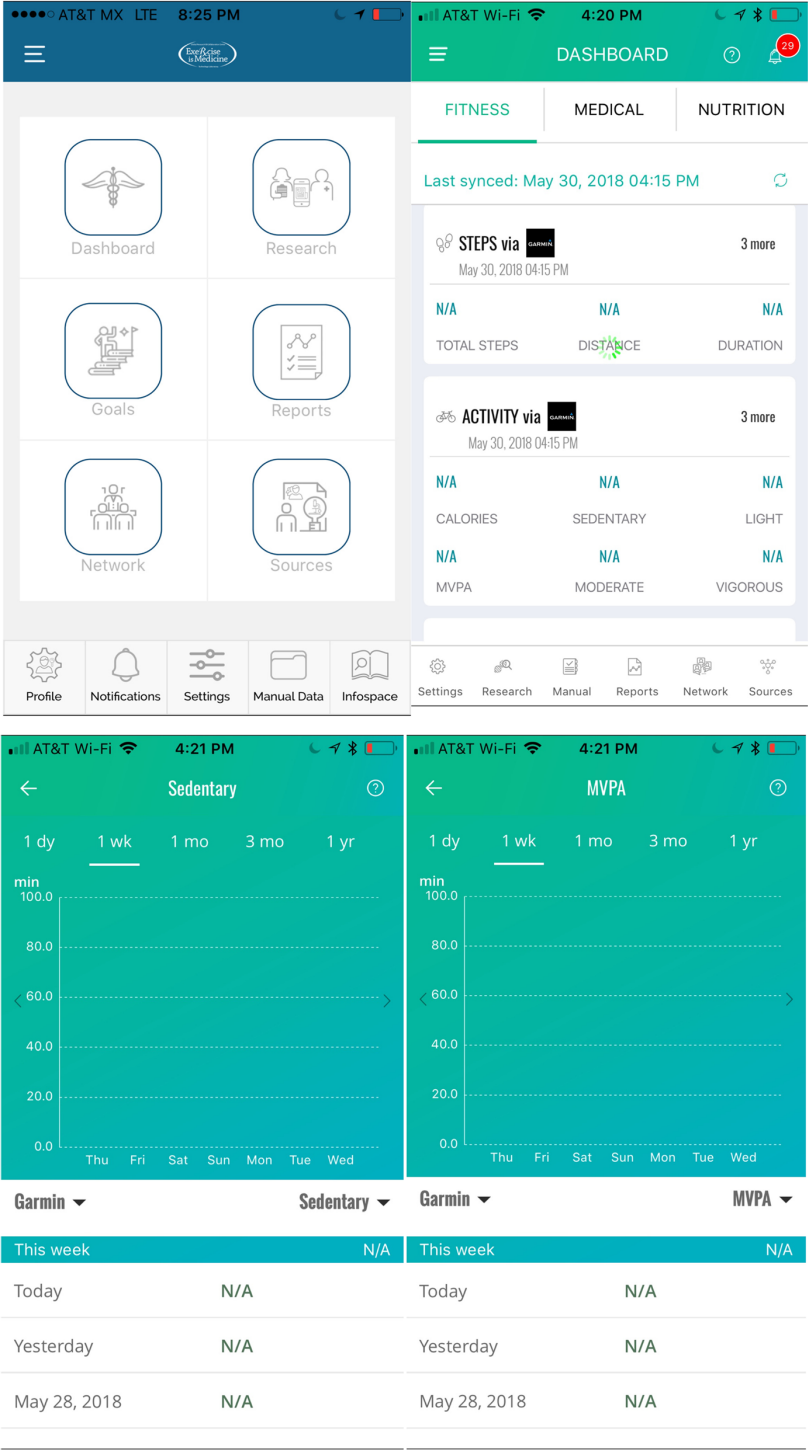
Integration of Exercise is Medicine framework into Quantextual Smartphone Application

In order to support behavior change, all participants will receive a consumer-oriented “wearable” an associated smart-phone app to enable continued monitoring of y sustained by study participants as part of the study’s PA intervention programming (Fig. 2). The Garmin Vivofit 3 PA monitor was selected based on device usability and provision of evidence-based behavior change techniques, as summarized by a systematic content analysis of behavior change strategies implemented in commercially available electronic activity monitors https://www.quantextual.co/researcher/, [43]. The Garmin Vivofit 3 measures and displays steps, calories, distance, minutes of activity intensity and sleep time. Furthermore, the wearable device links to a health IT HIPAA-certified platform (Quantextual Inc) https://www.quantextual.co/researcher/. that automatically syncs the user’s PA information and allows them to self-monitor their progress, evaluate goals, interact with EIM study staff and share results with fitness professionals and providers on a free custom smartphone application (IOS or Android). This App will also enable the study staff to remotely monitor participants PA engagement. As such, the wearable device can be considered a component of the intervention in addition to providing process data.

### Group 2

#### Group-based physical activity program

*In addition to the intervention components previously listed, group 2 participants will also be referred to group-based PA program.* We will procure an exercise space at each study site that is close to Nephrology Clinics and convenient for most participants to hold group-based exercise sessions. Before implementation, EIM fitness professionals will participate in a single 6-h training session conducted by one of the authors of the curriculum. The training consisted of an introduction and discussion of the curriculum objectives, demonstration of activities, and overview of the program’s “Move and learn” activities.

At each location, we will make available blood pressure cuffs, glucometers and glucose strips for persons who experience symptoms related to hypotension or hypoglycemia during the class. Program sessions (8 persons per group) will be specifically designed for persons with CKD. Sessions will offer progressive aerobic conditioning, muscular strength and endurance, flexibility, and stability, balance and will be led by certified EIM fitness professionals [44].

Fitness professionals will follow the 2008 PA guidelines safety recommendations for PA programming tailored to persons with CKD and will be trained on communication skills and behavioral techniques (goal-setting, action planning, self-monitoring, provide instruction, feedback on performance, opportunities for social comparison, peer modeling, and social support) [45]. Two sessions/week will be offered, slowly progressing towards 50 min of light-to-moderate PA per session. Persons will also be encouraged to accumulate additional minutes of moderate PA throughout the week, primarily through increased walking for leisure and transportation. Interventions are structured around “*Move and Learn*” formats. They will include health education, led PA and lifestyle behavior change segments modeled after evidence-based sources (interventions examples are shown in Table 3) [46]. The social dynamics of the group will be used to forge strong, sustainable relationships with participants and reach appropriate minutes per week of PA, collect data using Health IT resources and provide guidance at the end of 8-week core intervention for transitioning to maintenance and self-managed PA. We will continue to offer the intervention even if the the participant initiates dialysis. For particiapnts who recently underwent surgery for dialysis access, we will encourage participation but limit arm exercise.

**Table 3:** Study Procedures and measurements

	Baseline	Follow-up: 1	Follow-up: 2
		8 weeks	16 weeks
Self-reported			
Physical activity vital sign	√		
Medical history (or any changes thereof)	√	√	√
Quality of life (SF-12)	√	√	√
Qualitative Interview^a^	√		
Adverse events		√^b^	√
MEASUREMENTS			
Grip strength	√	√	√
6-min walk test	√	√	√
Anthropometry (measured BMI, waist circumference)	√	√	√
Blood pressure and blood glucose level	√	√	√
Weekly physical activity as measured by Garmin PA monitor	√	√^b^	√
INTERVENTIONS			
Brief physical activity counseling	√		
Wearable physical activity monitor	√	√	√
Group exercise sessions		√	

^a^in a subset of patients recruited at start of study

^b^assessed continually during the study in G2

## Measurements

Table 2 displays the data that we will collect during the study data. We plan to collect study data using Research Electronic Data Capture (REDCap) electronic data capture tools hosted at Emory University and Stanford University. REDCap is a secure, web-based application designed to support data capture for research studies providing 1) an intuitive interface for validated data entry; 2) audit trails for tracking data manipulation and export procedures; 3) automated export procedures for seamless data downloads to common statistical packages; and 4) procedures for importing data from external sources.

### Primary outcome

#### Minutes per week of physical activity

The Study main outcome will be assessed objectively using the Garmin vivofit werable device. Weekly minutes of moderate-to-vigorous PA will be synchronized every week using the Quantextual application. In case of problems we will also manually sync the werable devices at the V1 and V2 measurement time points. In addition, weekly number of steps, sleep hours and minutes of sedentary time will also be collected.

### Secondary outomes

#### Questionnaires

- The Center for Epidemiological Studies-Depression questionnaire (CES-D): CES-D20, is a 20-item instrument that asks individuals to rate how often over the past week they experienced symptoms associated with depression, such as restless sleep, poor appetite, and feeling lonely. Response options range from 0 to 3 for each item (0 = rarely, 1 = little of the time, 2 = much of the time, 3 = almost all the time). Scores range from 0 to 60, with high scores indicating greater depressive symptoms. The CES-D also provides cutoff scores (e.g., 16 or greater) that aid in identifying individuals at risk for clinical depression, with good sensitivity and specificity and high internal consistency [47]
- Short Form Health Survey (SF-12) [48]. The SF-12 questionnaire consists of 12 questions to measure functional health and wellbeing of the including a global question of perceived health status, to which the respondents indicate whether they think their overall health is excellent, very good, good, fair and poor. It covers the same eight health domains as the SF-36 with one or two questions per domain. The maximal number of points is 100 which in turn represents the best possible health condition and quality of life. SF-12 have been validated among dialysis patients and is in strong agreement with those derived from SF-36 in this population (*r* = 0.94 for both physical and mental component) [49].
- McAuley physical activity self-efficacy questionnaire [50]: The Exercise Self-Efficacy Scale assesses an individual’s beliefs in their ability to continue exercising on a three time per week basis at moderate intensities for 40+ minutes per session in the future. For each item, participants indicate their confidence to execute the behavior on a 100-point percentage scale comprised of 10-point increments, ranging from 0% (not at all confident) to 100% (highly confident). Total strength for each measure of self-efficacy is then calculated by summing the confidence ratings and dividing by the total number of items in the scale, resulting in a maximum possible efficacy score of 100.

#### 6-min walk test

All eligible participants will undergo 6-min walk test according to American Thoracic Society’s guideline [51]. In short, the test will be performed indoors on a 30 m straight course. Participants graded their dyspnea and fatigue perception on the 10-scale Borg scale. At the end of the 6-min period the distance, heart rate and blood pressure will be measured, and immediate fatigue and dyspnea scores will have reassessed. The participants will receive the same instructions before the walk and will be encouraged by the investigator who will repeat the set of phrases every 30s during the walk.

#### Grip strength

We will carry out an assessment of grip strength in a standardized manner i.e., in neutral position of arm, forearm, and wrist. We will measure three consecutive attempts (with one-minute intervals in between) with a digital dynamometer (Takei 5401; Takei Scientific Instruments, Inc). All measurements will be done on setting II as suggested by Firrell and Crain and recorded in kilograms [52] . The same dynamometer will be employed for all participants. Calibration will be done automatically (electronic zero calibration system). For each participant, the arithmetic mean value of 3 measurements with the dynamometer will be used for statistical analysis. This method shows high repeatability [53].

## Statistical analysis

### Sample size justification

The study is powered on between-arm differences in the proportion achieving the primary outcome, minutes per week of moderate-level PA. Our sample size estimation is guided by PA counseling and referral review studies report an effect size between 0.09 and 0.71 for achieving PA guidelines [54–58]. We used the formula given in Vierron and Giraudeau [59] and Vittinghoff and Fitzmaurice [60] with an adjustment for the intraclass correlation coefficient (ICC), where N is the total sample size, the Z’s are the critical value for type I (α = .05) and type II (β = .2) error, σ^2 is the variance in outcome, ρ is the ICC, and π is the proportion (50%) of the N persons assigned to the treatment group. Based on the above formula, assuming an ICC of 0.5 [61], our total sample size of 98 will provide 89% power to detect a 100 min/week of moderate PA difference between groups, corresponding to an effect size of 0.46. Considering an estimated 30% loss to long-term follow-up as reported in similar exercise intervention trials [62], our plan to enroll 98 participants should provide adequate power (80%) to detect differences across intervention arms. Data gathered from those who withdraw from the study will not be used for statistical analyses. This has been accounted for when completing the power analysis, with an additional 20% added to the target sample size. Attrition may be for a number of reasons, which will be ascertained upon withdrawal where possible.

## Expected outcome

Our primary outcomes is between-group difference in change in minutes/week of moderate-tovigorous PA between group 1 and 2 at 16 weeks. Based on our previous work, we expect greater improvement in grip strength, 6- min walk test, and PA among group 2 compared to group 1 participants. [54, 63]

### Quality assurance and trial coordination

We will implement a clear set of quality assurance steps for trial coordination and delivery of the intervention. Pre-specified recruitment criteria and protocols, rigorous training of personnel at both sites, and monitoring of sites with feedback will help assure high-quality study findings. In order to deliver the multi-level PA intervention strategy uniformly across sites, we will conduct training and certification, provide standardized tools, conduct site-initiation visits, team meetings, and study staff role into each existing patient flow. Data will be collected using standardized tools and instruments. All safety concerns, acute events [e.g., myocardial infarction injuries], or mortality noted during 16-weeks of trial follow-up will be compared between study arms and reported to oversight committees.

#### Data analysis

Principal Investigators (Stanford University and Emory University) will be given access to the cleaned data sets. Project data sets will be housed on the REDCap hosted by the respective universities and all data sets will be password protected. To ensure confidentiality, data dispersed to project team members will be blinded of any identifying participant information.

Between-group differences in primary and secondary outcomes will be assessed at 8 and 16 weeks. We will test for differences in primary and secondary outcomes to assess treatment effects using multivariate regression models adjusting for baseline characteristics. We will use mixed-effects linear and logistic regression models to predict changes in continuous PA levels and probability of achieving PA recommendations by regressing study arm, study centers, and an interaction between study arm*centers. The mixed model will include one random effect term accounting for the correlation within study center. Analysis of effectiveness will be according to an intention-to-treat analysis including all enrolled and randomized persons into our analysis. Irrespective of compliance with treatment, all participants will be encouraged to continue with the scheduled outcome evaluation until the end of the study. However, as high as up to 30% loss to long-term follow-up would be expected. The characteristics for those participants without complete follow-up will be examined. If missing data is determined to be random, we will perform a sensitivity analysis using multiple imputation-based methods. If missing observations cannot be assumed to be random, pattern mixture models will be considered [64].

#### Potential risks

Adverse effects of exercise: Minor risks include muscle soreness, light-headedness/dizziness, and weakness. Physical activity may lead to more serious health problems such as a heart attack and sudden death. However, this is very rare. Estimates of sudden cardiac death range from 0 to 2 per 100,000 h of physical activity. Physical Activity may also lead to acute spikes in blood pressure and/or blood glucose if the patient is not well-controlled. Group exercise classes will be led by trained professionals who are able to offer suggestions to limit and prevent these adverse events. Session will also include measurements of blood pressure and glucose to identify potential contra-indications for exercise that day. Fitness professionals will be trained to recognize medical emergencies and seek emergency care if needed. A potential participant’s nephrologist will be informed of their patient’s possible participation in our trial and if they determine a patient is medically unfit to participate, they will be excluded from the study.

Participants will be informed at each visit and exercise session that if at any point during the study they develop chest pain, shortness of breath, lightheadedness, or fall they should stop activity immediately and inform study staff if currently at a study visit or call 911 if at home. In the event of milder symptoms at home, they are advised to call study coordinators to inform them of their symptoms as they may alter future intervention. Participants’ nephrologist will be notified of all events and reported symptoms and will review the case before study should be continues.

#### Loss of confidentiality

Participation in the study will mean that some protected health information will need to be obtained from study participants. This information will only be shared with persons involved in the study and listed on the informed consent form. All information will be de-identified if used for other purposes such as medical publications.

#### Trial status

Study is currently ongoing and recruitment has begun at Stanford University site. Inclusion is expected to be complete by early 2019.

## Discussion

Physical inactivity is a major problem in persons with advanced CKD and transitioning to dialysis and a modifiable risk factor for many important outcomes including function, quality of life, and mortality. Several attempts have been made to make exercise more convenient for CKD patients by different intervention modalities such as intradialytic exercise programs using cycle ergometers [21] and various resistance exercise programs [65]. Despite these attempts at tailored interventions, patients are not adhering to exercise regimens. When Konstantinidou et al. [66] compared intradialytic exercise, home based exercise, and rehabilitation center-based exercise, the group that participated in the intradialytic exercise program had a dropout rate of 16.7%, as compared to 23.8% dropout rate for patients in the rehabilitation center-based group and 16.7% in the home-based exercise group.

In order for clinicians and health care systems to create tailored exercise interventions that promote exercise adherence, further examination of barriers to and motivators for exercise in CKD patients is needed. Our interdisciplinary approach includes vital input from the target populations, fosters physician-patient interactions around this topic, and is based on use of existing clinical and community resources, supplemented with trained EIM fitness professionals for whom we will develop a curriculum specific to persons with advanced kidney disease. If shown to be efficacious and effectively implemented, our research will be translatable immediately within the two major practices involved in the study and with modest effort, to other practices within the region.

Our previous “proof of principle” feasibility study which compared the effectiveness of the supplemented EIM protocol (PA vital sign + exercise referral arm) compared to the EIM protocol alone (PA vital sign) in the general population demonstrated a greater improvement in PA among individuals in the referal group (changes in self-reported PA: rerefal group: + 250 min/week vs.

EIM arm: − 38.6 min/week; *p* = 0.0002) [67]. Our group also evaluated the effectiveness of a PA referral program analogous to the EIM framework for hypertensive adults in Mexico’s Social Security healthcare system and found this strategy was well accepted by practitioners and particiapnts, with significant improvements in moderate-to-vigorous PA minutes reductions in diastolic blood pressure (4.5 mmHg), waist circumference (2.5 cm) and fasting blood glucose (5 mg/dl) all *p* < 0.05 in the referral group compared to usual care [63, 68]. Therefore, the EIM framework has a track record for feasibility of implementation in clinical settings with evident gains in PA among participants especially when referal to group-based exercise sessions led by certified fitness professionals We believe this pragmatic approach has great potential to engage nephrologists and patients with advanced CKD in positive, motivational interactions with specific, well-designed follow through plans.

This study will be the first to assess the EIM framework and additional exercise session referral strategy in the CKD population. This will allow for potential application of this protocol in other nephrology practices with little clinical burden on existing staff. A further strength of our study is the adaptation of this program in two racially and ethnically diverse areas. In addition, the intervention is built on proven science and tailored to the unique needs of the study population as assessed in a series of formative, in-depth interviews. The limitations of this study include potential selection bias of highly motivated persons within nephrology practices, so effects may not be generalizable to the entire CKD population. Further, due to the short duration of the study, long-term outcomes such as mortality or delays in start of dialysis cannot be obtained with sufficient power.

Once complete, we hope to adapt the main intervention according to the successes and challenges of the initial trial. We will contact dialysis organizations (large or small) and seek individual meetings with administrators at dialysis and insurance (i.e Medicare, private) organizations to create a larger roll out. Working with the ACSM we can easily disseminate the personnel training and equipment used in the intervention as a package for larger implementation in the US and internationally as part of the Global EIM initiative.

## Conclusion

In sum, the current feasibility trial will be an important step towards an important and urgently-needed intervention for patients on dialysis who are extremely sedentary. We will employ the strengths of the health systems and clinicians to initiate PA promotion and referrals to community programming. We will supplement existing community resources by providing trained and “linked-in” EIM practitioners with specific knowledge of the challenges facing patients transitioning to dialysis. We plan to inform the development of a pragmatic, cost-conscious and scalable intervention ‘package’ that facilitates integration of PA into the routine care of CKD clinical populations.

## Abbreviations

ACSM: American College of Sports Medicine
CES-D: Center for Epidemiological Studies-Depression questionnaire
CKD: Chronic Kidney Diseases
EIM: Exercise is Medicine
PA: Physical activity
SF12: Short Form Health Survey
